# Clinicopathological Significance and Expression Pattern of Bcl2 in Breast Cancer: A Comprehensive *in silico* and *in vitro* Study

**DOI:** 10.1016/j.sjbs.2023.103916

**Published:** 2023-12-23

**Authors:** Shazia sofi, Umar Mehraj, Nusrat Jan, Abdullah Almilaibary, Irshad Ahmad, Fuzail Ahmad, Manzoor Ahmad Mir

**Affiliations:** a Department of Bioresources, School of Biological Sciences, University of Kashmir, Srinagar 190006, India; b Department of pathology, Duke University, Durham, NC 27708, United States; c Department of Family & Community Medicine, Faculty of Medicine, Al Baha University, Albaha 65511, Saudi Arabia; d Department of Medical Rehabilitation Sciences, CAMS, King Khalid University, Abha, Saudi Arabia; e College of Applied Sciences, Almaarefa University, Diriya, Riyadh 13713, Saudi Arabia

**Keywords:** Chemoresistance, Breast Cancer, Metastasis, Apoptosis, B cell lymphoma gene 2, Oncogene, Expression pattern

## Abstract

B-cell lymphoma/leukemia gene-2 (Bcl-2) is the primary proto-oncogene that has been shown to work by preventing apoptosis/programmed cell death. Bcl-2 combines a variety of cell-generated signals associated to the survival and death of cells. In glioma, lung, and breast cancer, Bcl-2 over-expression has been linked to an increase in invasion and migration. Many treatment regimens that target Bcl2 have been established and approved, and thus increasing the survival rates of the patients. The primary goal of this research was to recognize new therapeutic compounds that target Bcl2 and assess Bcl2 expression pattern in BC patients. We used various bioinformatic tools as well as several *in vitro* assays to look out the expression and inhibition of Bcl2 in BC. Our study depicted that Bcl2 had a strong connection with tumour stroma, notably with suppressor cells originating from myeloid tissues. Moreover, *in vitro* and *in silico* research identified Paclitaxel as a promising natural substance that targets Bcl2. Overall, this work shows that Bcl2 overexpression accelerates the development of BC, and that targeting Bcl2 in combination with other drugs will dramatically improve BC patient’s response to treatment and prevent the emergence of drug resistance.

## Highlights

• This study reflects the pivotal role and prognostic consequence of Bcl2 in BC pathogenesis.
• Bcl2 was selected as a potent target for this study, as its overexpression accelerates the Breast tumorigenesis.
• In-vitro and in-silico outcomes reveal that Bcl2 enhance tumorigenesis and exhibit worse clinical outcomes.
• Targeting BCL2 in combination with other therapeutic drugs will dramatically improve Breast cancer patients' responses to therapy and prevent the emergence of chemoresistance.
• Bcl2 acts as a prognostic marker and antagonizing it could be one of the potential therapeutic strategies to treat BC.

## Graphical abstract

**Figure:**
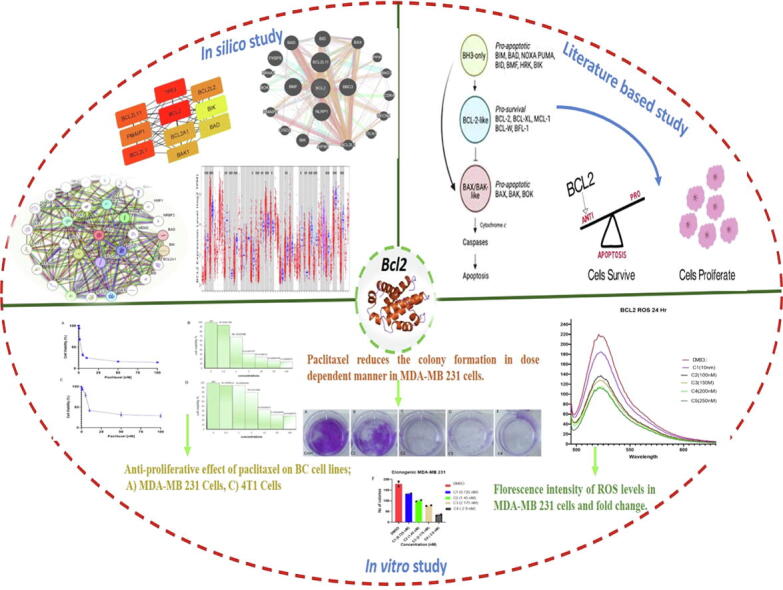

## 1 Introduction

With about 25 % of all female malignancies, BC is the most widespread type of malignancy globally (Sung et al., 2021, Mehraj et al., 2022). Males can also get BC, even though women are 100 times more likely to do so (Fundytus et al., 2020, Mehraj et al., 2022). BC is the cause of about 15 % of all tumour-related deaths in females (Qayoom et al., 2021). Sadly, the prevalence of this type of cancer has recently surged in emerging countries, where it tragically affects a staggering number of women (Mehrotra and Yadav, 2022). In India, one in every 29 women will develop BC (Mehraj et al., 2022, Yousuf et al., 2022). One-fifth of all malignancies among women in India and 7 % of the global burden of breast cancer are caused by it (Monica and Mishra, 2020). Epidemiological studies predict that there will be more than two million cases of BC worldwide by the year 2030 (Mehraj et al., 2022, Torres-Román et al., 2023). In urban regions, the incidence of breast tumor is nearly three times higher than in rural areas. For 100,000 women, the age-standardized incidence rates vary from 9 to 32 (Lei et al., 2021). Breast cancer is often treated as a group of diseases due to the occurrence of numerous biological subgroups (>100) with distinct molecular profiles and clinicopathological characteristics (Qayoom et al., 2023). The distinction between receptor-positive (Normal like, Luminal A, Luminal B, and HER-2 positive) and receptor-negative (TNBC or basal like) subtypes of breast cancer has been made using gene expression profiling, in addition to histological subtypes (Kakudji et al., 2021). Among its molecular subtypes, TNBC is the utmost aggressive BC subtype (Sofi et al., 2022). Due to its distinct molecular makeup, TNBC is not amenable to molecular targeted therapy or endocrine therapy (Xu et al., 2022). Chemotherapy is therefore the main systemic therapy, although adjuvant chemoradiotherapy given postoperatively is unsuccessful (Mehraj et al., 2022, Sofi et al., 2023).

The hallmarks of cancer including angiogenesis, uncontrolled growth, and apoptosis evasion exist in all tumor cells regardless of the cause or type (Meliala et al., 2020, Sofi et al., 2022). One of the main aim of apoptosis is the retardation of cancer growth. The process of “programmed cell death,” sometimes referred to as “apoptosis,” which is a response to environmental stimuli, is useful for the treatment and inhibition of cancer (Tompkins and Thorburn, 2019). The key players in the process of regulation of apoptosis include the Bcl-2 family. The Bcl-2 family includes both pro-apoptotic and anti-apoptotic proteins, which activate or inhibit the apoptotic program accordingly. A slight change in the dynamic balance of these proteins may result either in inhibition or promotion of cell death (Rahman et al., 2022). Solid tumours and hematological neoplasias are among the numerous disorders for which Bcl2 is a significant target for therapy due to the important role it plays in tumorigenesis and treatment resistance (Ploumaki et al., 2023). Numerous anticancer medications target the Bcl-2 family because it is crucial to cell survival or death. Recent developments in medicine have made it possible to specifically target Bcl-2 family members (Alam et al., 2021). (Zhang et al., 2020, Qayoom et al., 2021).

The first proto-oncogene to have a role in suppressing apoptosis and programmed cell death has been identified as the Bcl-2 gene (Zheng et al., 2020). Multiple cell-generated signals related to survival and death are generated by Bcl-2. However, the mechanism behind the anti-apoptotic feature of Bcl-2 is still poorly understood (Czabotar and Garcia-Saez, 2023). Prior research revealed that Bcl-2 leads to the invasion and migration of malignant cells (Denisenko et al., 2019). In lung, glioma, and BC cells, it has been detected that overexpressing Bcl-2 accelerates migration and invasion (Um, 2016). Kang et al. verified in animal models that the metastasis of BC to the bone was caused by the overexpression of Bcl-2 (Kang et al., 2003). Following this, it was shown that Bcl-2 causes the mouse EpH4 mammary epithelial cell line to undergo cellular metastasis to the lung (Pinkas et al., 2004). Research efforts to create chemical Bcl2 inhibitors have been widely documented. Around 75 % of initial breast cancer cases exhibit increased levels of Bcl-2, with 85 % of these tumours expressing oestrogen receptor (ER) positivity and 50 % expressing HER-2 (Tawfik et al., 2012). A high level of Bcl-2 seems to be related with a worse clinical outcome in cases of BC (da Silva Lawisch et al., 2022). According to the examination of patient samples, breast and colorectal cancer liver metastasis was linked to Bcl-2 expression in tumor cells (Zhang et al., 2020). Additionally, in lung cancer, the expression of Bcl2 was observed to get upregulated during the transition from pre-invasive to invasive stage (D’Aguanno and Del Bufalo, 2020). Normal cells are not significantly affected by Bcl-2 protein inhibitors because tumour cells express the protein at far higher levels than normal cells do. Thus, a novel therapy regimen based on tumour etiology involves blocking the Bcl2 anti-apoptotic protein to overcome the tumour cells' resistance to apoptosis (Qian et al., 2022). In this context, many drugs targeting Bcl2 have been studied for their anti-inhibitory role against this anti-apoptotic and proto-oncogene Bcl2(Perini et al., 2018). One of the natural compounds that target this anti-apoptotic Bcl2 is Paclitaxel. Paclitaxel (PTX) belongs to a taxane family and is an antineoplastic drug that affects the stability of microtubules and is frequently employed as a chemotherapeutic agent in various tumours (Maloney et al., 2020, Mosca et al., 2021). PTX has been shown to have antimitotic properties in numerous investigations (Sousa-Pimenta et al., 2023). Additionally, there are various levels of mechanism of action for the PTX action linked to the prevention of tumour growth (Alqahtani et al., 2019). According to studies, paclitaxel started a chain reaction of signalling pathways that led to programmed cell death (Hongmei, 2012).

We used a bioinformatics approach in this study to examine Bcl2 expression and its predictive value in Breast cancer (BC). The prognostic relevance, expression pattern, genetic modification, and immunological connection of Bcl2 in the diverse breast cancer subtypes were investigated using the tools GEPIA, Bc GeneExminer, UALCAN, TIMER 2.0, ENRICHR, and TISCH. Further *in vitro* screening was done along with *in silico* testing. A high expression of Bcl2 in BC patients shows worse clinical outcomes, which indicates that BCL2 is essential for breast tumorigenicity (Honma et al., 2015). This study will also focus on targeting Bcl2 with Paclitaxel which will considerably improve the way BC patients respond to treatment and lower the emergence of chemoresistance. Therefore, our study will primarily focus on both the Invitro and Insilco parameters for targeting the highly dysregulated Bcl2 in breast cancer.

## 2 Material and methods

### 2.1 Expression profile analysis of Bcl2 in PAN cancer

The vast online resource TIMER 2.0 portal is applied to investigate immune infiltrates and expression patterns of genes in a variety of cancer types (https://timer.cistrome.org/) (Li et al., 2020). This portal was applied to study the Bcl2 expression in diverse cancers using TCGA information.

### 2.2 UALCAN

To assess Bcl2 expression in various BC subclasses, ethnicities, and age ranges of BC patients, we used the UALCAN database, an inclusive online reservoir for evaluating cancer OMICS data (Chandrashekar et al., 2017). With coupled proteomic, whole-exome, and CNA data, UALCAN was also utilized to evaluate pathway-level somatic mutations in Bcl2 across tumours, involving important pathways for various cancer types.

### 2.3 bc-GenEXMiner

The link between Bcl2 expression and other clinicopathological traits of BC patients, such as hormonal status, nodal status profile, SBR grade, and p53 status, was investigated using BC Gene-Expression Miner V4, a web-based bioinformatics tool for annotated BC transcriptomic data (Jézéquel et al., 2012).

### 2.4 GeneMANIA (https://genemania.org/)

GeneMANIA provides supplementary set of genes that are linked to a group of given genes using a vast database of functional association data. (Warde-Farley et al., 2010). Protein and genetic connections, pathways, protein domain similarity, co-expression, and co-localization are a few examples of association data. We predicted Bcl2's function and relationships between genes using this online tool GeneMANIA. The function of all interactive genes can all be predicted using this adaptable and user-friendly online database. The database was utilized to forecast Bcl2′s function and relationship to other important genes.

### 2.5 Bcl2 analysis in single-cell sequencing database

To see severely altered Bcl2 expression across different datasets in BC tissues, the Tumor Immune Single-cell Hub (TISCH) was initially processed (Sun et al., 2021). We studied Bcl2 expression in primary and metastatic BC datasets to depict the association between Bcl2 expression and tumour development.

### 2.6 Analyzing the protein–protein interaction (PPI) of Bcl2

A Bcl2 PPI network was created using STRING database (https://stringdb.org) (v-11) (confidence score value of 0.7). A biological web resource called STRING was created to create and evaluate functional protein connections. The Cytoscape tool was used to further analyse and envision the PPI web (v-3.8.2). The distinct modules of the PPI web were obtained using the MCODE plug-in. Using the cytohubba plugin option, top 10 HUB proteins in the PPI web were identified (Szklarczyk et al., 2015).

### 2.7 Pathway enrichment and gene ontology (GO) of Bcl2

A significant database called GO aims to link the illustration of gene and its product properties across all the species. It provides us with a gradually assembled collection of many of the systematized terminologies for cellular constituents, molecular processes, and biological functions. The GO annotation study was evaluated by Enricher, an intensive online resource that gathers biological knowledge for additional biological discoveries. It was also utilized to look at the KEGG pathway analysis (Kanehisa et al., 2017).

### 2.8 Docking of paclitaxel and Bcl2

#### 2.8.1 Selection and preparation of target

The target protein, Bcl2, was downloaded in PDB style from the PDB with ID 6GL8 (Birkinshaw et al., 2019). The 'Prepare protein' module in DS was utilized to normalize atom names, get rid of disorderly alternate conformations, add main- and side-chain atoms that are missing, and reduce the structure for Bcl2. Heteroatoms and water molecules were also taken out.

#### 2.8.2 Molecular docking

The objective of this research is to elucidate the binding affinities of the compound paclitaxel for Bcl2 protein. Docking analyses were conducted using Autodock v 4.2.6 for the compound of interest. The pre-established co-crystallized X-ray structure, sourced from the RCSB PDB, (6GL8) facilitated the determination of protein binding cavities. Residue locations within a 3 Å radius were computed utilizing the co-crystallized ligand. During the cavity selection phase, Chimera software (https://www.cgl.ucsf.edu/chimera/) was employed to eliminate co-crystallized ligands, followed by energy minimization via steepest descent and conjugate gradient methods. Subsequently, both the receptor and target compound were saved in pdbqt format after merging non-polar hydrogens. Molecular docking was executed within a grid box of dimensions 18 x 15 x 17 Å and 0.3 Å spacing. Protein-ligand complex docking experiments adhered to the Lamarckian Genetic Algorithm (LGA) framework. Three separate molecular docking trials were conducted, each consisting of 50 solutions, a population size of 500, 2,500,000 evaluations, and a maximum generational number of 27, while maintaining default settings for all other parameters. Upon completion of the docking process, RMSD clustering maps were generated by re-clustering using clustering tolerances of 0.25, 0.50, and 1 to identify the optimal cluster with the lowest energy score and highest population.

#### 2.8.3 Molecular dynamics simulation

Using the Desmond 2020.1(Shaw et al., 2010), MD simulations were done on paclitaxel + Bcl2 complex. At different temperatures of 50, 60, 70, 80, 90, and 100°Celsius, models were run separately for each complex and the reference (Shivakumar et al., 2010). In this system, the OPLS-2005 force field and explicit solvent model with the SPC water molecules (Jorgensen et al., 1983) were used in a period boundary salvation box with dimensions of 1.0 Å x 1.0 Å x 1.0 Å. In order to neutralise the electrical charge, sodium ions (Na + ) were introduced. The metabolic environment was replicated by introducing a solution containing 0.15 M NaCl into the apparatus. Initially, the system underwent retraining by subjecting the protein–ligand complexes to an NVT ensemble for a duration of 10 ns. Following the preceding procedure, an NPT ensemble was employed to conduct a brief 12-ns run of equilibration and minimization. The NPT ensemble was established using the Nose-Hoover chain coupling method (Martyna et al., 1994). In each of the models, the temperature was varied while maintaining a constant relaxation time of 1.0 picoseconds and a pressure of 1 bar. The temporal increment utilised in the simulation was 2 fs. The Martyna-Tuckerman-Klein chain coupling scheme barostat technique (Martyna et al., 1992) was employed to regulate the pressure. The period of relaxation was chosen as 2 picoseconds. In the computation of long-range electrostatic interactions, we employed the particle mesh Ewald approach (Toukmaji and Board Jr, 1996). The radius for the coulomb interactions was maintained at a constant value of 9 Å. The bonded forces for each trajectory were calculated using the RESPA integrator with a time step of 2 fs. The most recent manufacturing run was conducted with a temporal duration of one hundred nanoseconds per unit. To assess the stability of the molecular dynamics (MD) simulations, several key parameters were quantified, including the root mean square deviation (RMSD), radius of gyration (Rg), root mean square fluctuation (RMSF), and number of hydrogen bonds (H-bonds). The aforementioned values were employed to assess the stability of the molecular dynamics (MD) simulations.

### 2.9 MTT assay

In a 96-well plate, BC cell lines 4 T1 and MDA-MB-231 at the seeding density of 3x10^3^ cells per well were seeded. The cells were given various doses of PTX after 24 h, and they were then incubated for 72 h in a humidified CO2 incubator. The MTT test was carried out using the MTT kit (Invitrogen) after 72 h. The manufacturer's procedure was followed when conducting the assay (Van Meerloo et al., 2011).

### 2.10 Colony formation assay

BC cells MDA-MB-231 were seeded at a seeding density of 1,000–1,500 cells/well in 6-well plates to observe the effect of paclitaxel on colony formation of BC cells. After 48 hours, the media was changed for a new one that included therapeutic supplements. The experiment took 14–18 days. Every 3 days, the therapeutic medium was replaced, and colonies were viewed in the wells by means of an inverted microscope. Once enough colonies had grown, they were dyed with crystal violet and fixed with 3.7 % paraformaldehyde. The ImageJ application was used to take pictures of the plates and count the colonies (Rajendran and Jain, 2018).

### 2.11 DCFDA assay

BC cells were seeded in 12-well culture plates and subjected to 24 h of paclitaxel treatment at various doses. The cells were then stained for 30 mins in the dark with 10-M 2′,7′-dichlorofluorescin diacetate (DCFDA) from Sigma-Aldrich, and the fluorescence was gauged using a fluorometer (Degli Esposti, 2002).

### 2.12 Statistical analysis

The statistical significance was analyzed using the one-way or two-way ANOVA in GraphPad Prism V 8.43, followed by Tukey multiple comparisons test. *p* < 0.05 was considered significant.

## 3 Results

### 3.1 Bcl2 is highly overexpressed in many malignancies

**Fig. 1:**
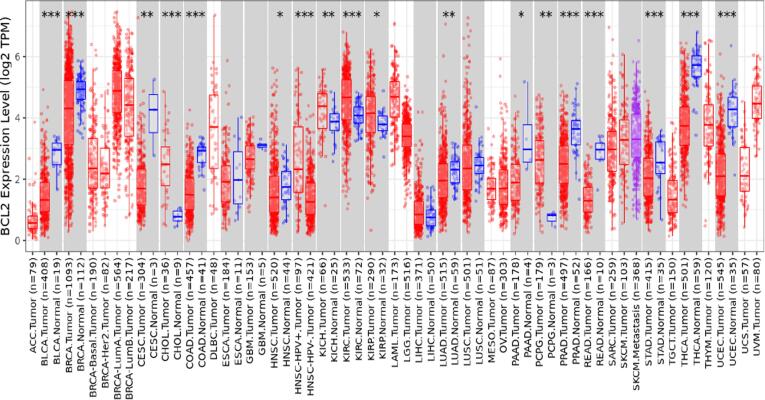
Expression of Bcl2 in several malignancies using box plot model form TIMER Database. Bcl2 is vastly upregulated in most of the cancers, especially breast cancer where it shows highest expression with respect to other tumors.

The TIMER 2.0 analysis showed that Bcl2 is considerably enhanced in numerous malignancies. The box plots reflected that Bcl2 is highly upregulated in many tumors, including LUSC, BC, KIRC, LGG, THCA, HNSC, SARC, LUAD, and UCEC **(**Fig. 1**).** The highest expression of Bcl2 among these cancers was seen in breast cancer patients.

### 3.2 Bcl2 overexpression positively associates with ER^+^ and PR^+^ status

**Fig. 2:**
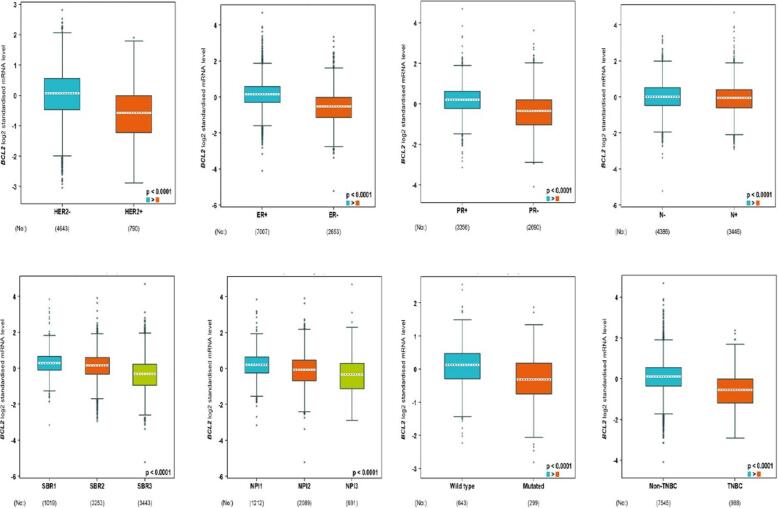
Bcl2 expression is linked with several clinicopathological features. The high expression of Bcl2 is associated with HER2^-^, ER^+^, PR^+^, Nodal negative, SBR1, NPI1, wild type P53, and non TNBC status.

The bc-GenEXMiner portal analyzed the link between Bcl2 and clinicopathological traits in BC patients. Bcl2 had a strong correlation with patients who lacked the HER2 receptor. Bcl2 is typically expressed at higher levels in BC patients with positive oestrogen and progesterone status. Additionally, Bcl2 expression levels in patients with nodal negative status show higher levels as compared to the nodal positive patients. Also, Bcl2 had strong expression in the SBR1 and NPI1 grades. Further investigation into the relationship between Bcl2 and p53 mutation status revealed that BC p53-wild type tumours typically exhibit high quantities of the Bcl2 gene**.** We also investigated the link between Bcl2 expression and TNBC profile in BC patients, and we discovered that Bcl2 is significantly overexpressed in tumours that are having non TNBC status (p-value of 0.0001) **(**Fig. 2**).**

### 3.3 Bcl2 highly correlates with BCL2L11

**Fig. 3:**
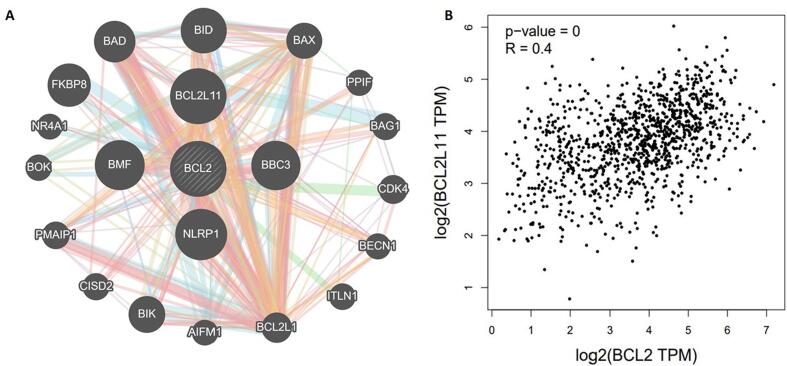
Gene-Gene interaction of Bcl2 with other genes show that Bcl2 is highly correlated with Bcl2L11, FKBP8, BBC3 and other genes. Correlation of Bcl2 with Bcl2L11 with a R value of 0.4 using GEPIA.

Using the GeneMANIA portal, we investigated the relationships between the genes in the Bcl2 gene network. Bcl2 showed significant interactions with BCL2L11 followed by NLRP1, BMF, BBC3, FKBP8, BAX, BAD, BID and quite a few other genes as shown in Fig. 3**A.** The network interaction of genes was based on physical connections, co-expression, co-localization, and genetic interaction pathways. Using Gepia2, the relationship between Bcl2 and BCL2L11 was investigated. The findings showed that Bcl2 and BCL2L11 have a strong correlation in BC patients, with a R value of 0.4 **(**Fig. 3**B)**.

### 3.4 Expression analysis of Bcl2

**Fig. 4:**
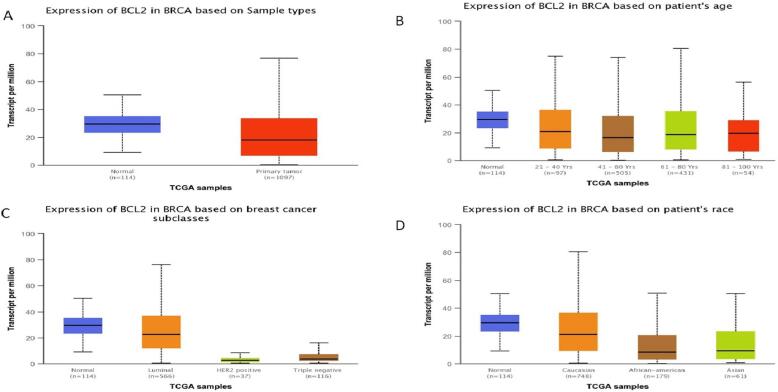
Expression profile of Bcl2 in BC patients. High expression was seen in Patients with primary tumor in the age group of 61–80. The expression was seen higher in Luminal BC patients and that too in the race of caucassian group.

Using the UALCAN database, expression analysis of Bcl2 about sample type, age group, BC subtypes, and ethnicity was further investigated. The analysis revealed that Bcl2 is overexpressed in primary tumor as compared to the normal patients. According to expression analyses, Caucasian women among the other races had elevated Bcl2 expression. The females under the age group of 61–80 show augmented expression of Bcl2. In addition, luminal patients displayed increased Bcl2 expression in comparison to HER2 or TNBC subtypes **(**Fig. 4**).**

**Fig. 5:**
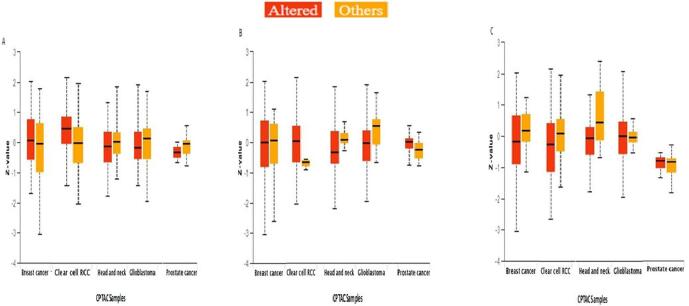
Pathway status in association with expression of Bcl2 across pan-cancer. The Bcl2 expression was examined for modulation in signaling pathways through, **A** Wnt signaling, **B** mTOR pathway, and **C** p53/Rb-related pathway (p-value < 0.01).

Additionally, using combined proteomic, CNA data, and whole-exome, we systematically examined pathway-level somatic alterations in Bcl2, implicating vital pathways across various cancer types. It was discovered that WNT signalling, mTOR signalling, and the p53-Rb pathway are all altered in cancer patients with an overexpression of Bcl2 **(**Fig. 5**).**

### 3.5 Association of Bcl2 with tumor stroma

**Fig. 6:**
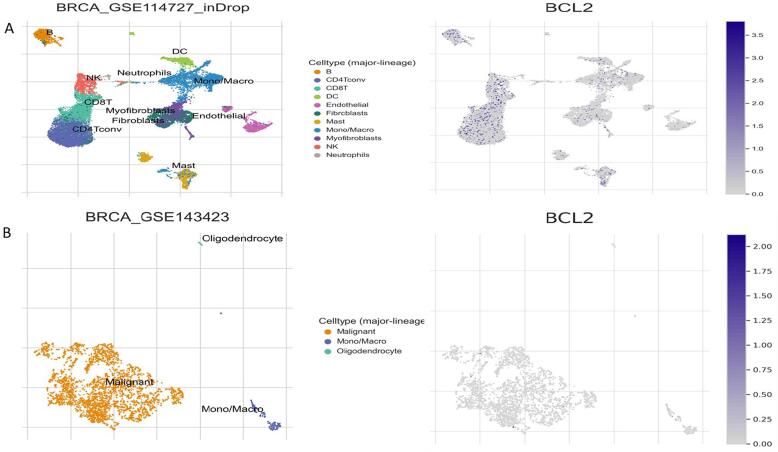
Expression pattern of Bcl2 in BC. A: Primary breast tumors. B: Metastatic BC. Bcl2 is expressed more in primary as related to metastatic tumors.

We assessed the expression status of deregulated Bcl2 in TISCH database, that focuses on TME. We examined expression profile of Bcl2 in few primary and metastatic tumor BC datasets. The analysis reflected that Bcl2 is expressed more in primary as related to metastatic tumors. Also, Bcl2 expression displayed diversity in expression profile across the primary tumor cell population. CD4T and CD8T cells presented increased expression of Bcl2 in BC individuals with primary tumors **(**Fig. 6**).**

### 3.6 PPI (Protein-protein interaction) of Bcl2

**Fig. 7:**
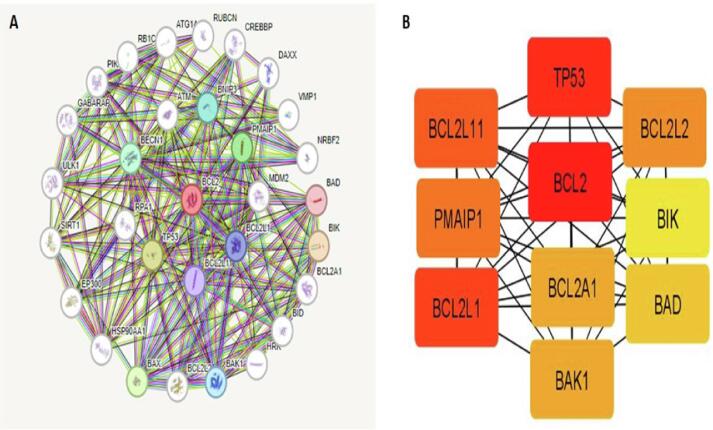
A) PPI interaction of Bcl2 with other proteins. B) Top ten hub genes obtained from PPI network using cytoscape.

STRING was used to create PPI by connecting 31 proteins (nodes) and 219 protein–protein interactions (edges). According to the PPI, average local clustering coefficient value of 0.789, the PPI enrichment p-value of < 1.0e-16, and average node degree value of 14.1 was obtained **(**Fig. 7**A).** Using Cytohubba, the top 10 genes based on degree score value were identified **(**Fig. 7**B).** Bcl2, Bcl2L11, TP53, PMAIP1, BCL2L1, Bcl2L2, Bcl2A1, BIK, BAD, and BAK1 were among the top genes in this list. The most vital elements of the PPI network were found by analysis of the MCODE plugin option in Cytoscape.

### 3.7 Pathway enrichment and GO analysis of Bcl2

**Fig. 8:**
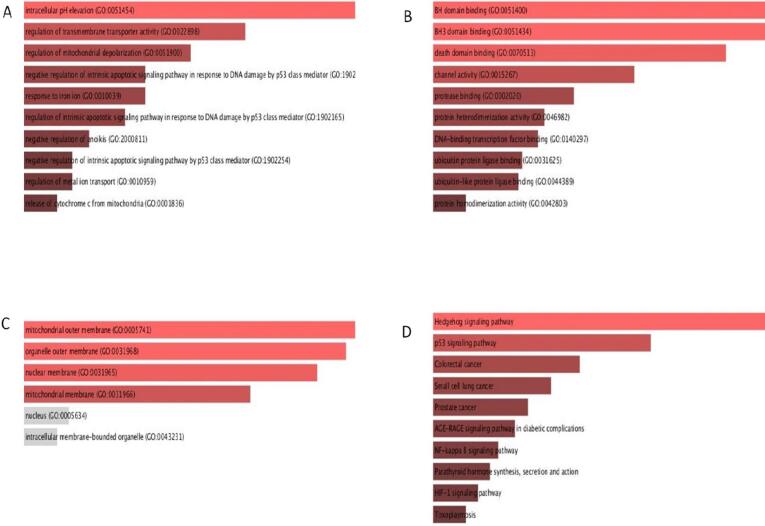
GO and KEGG analysis of Bcl2 in breast cancer.

Pathway enrichment and GO analysis were completed using the Enrichr portal. Among the MF, Bcl2 was involved in intracellular pH elevation, mitochondrial depolarization, regulation of transmembrane transporter activity, negative regulation of enoikis, etc. **(**Fig. 8**A).** Among the BP, Bcl2 was found associated with the protease binding, BH domain binding, death domain binding, DNA-binding transcription factor binding, protein heterodimerization activity etc. **(**Fig. 8**B).** Among the cellular compartment Bcl2 was highly enriched in outer membrane of mitochondria, and nuclear membrane **(**Fig. 8**C).** The KEGG pathway depicted that Bcl2 is related with Hedgehog signaling pathway, p53 signalling pathway, prostate cancer, small cell lung cancer, age, colorectal cancer, race, signalling pathway in diabetic complications, NF-κ β signalling pathway **(**Fig. 8**D).**

### 3.8 Molecular docking displayed significant binding of paclitaxel with Bcl2

**Fig. 9:**
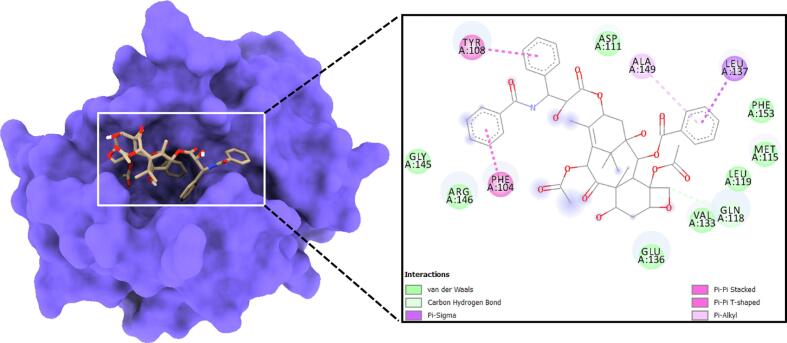
Molecular surface view of the Bcl2 with paclitaxel bound in deep cavity. 2D interaction exhibiting the interactions between ligand and protein and dotted lines exhibiting interactions.

All the binding energy scores are calculated from the best cluster (95 %) that falls within the lowest RMSD 0.25 Å. With the lowest binding energy between paclitaxel and BCL2, showed a considerable binding affinity ΔG – 7.2 kcal/mol. During the interaction, the paclitaxel and Bcl2 exhibited no conventional hydrogen bonds, except carbon hydrogen bond with Gln118, pi-sigma pair interaction with Leu137, pi-pi stacked Phe104, Tyr108 and pi-alkyl Ala149 residues (Fig. 1). Other non bonded interaction such as vander Waal’s was formed with the residues exhibited floating in then 2D interaction plot **(**Fig. 9**)**.

### 3.9 Molecular dynamics simulation

**Fig. 10:**
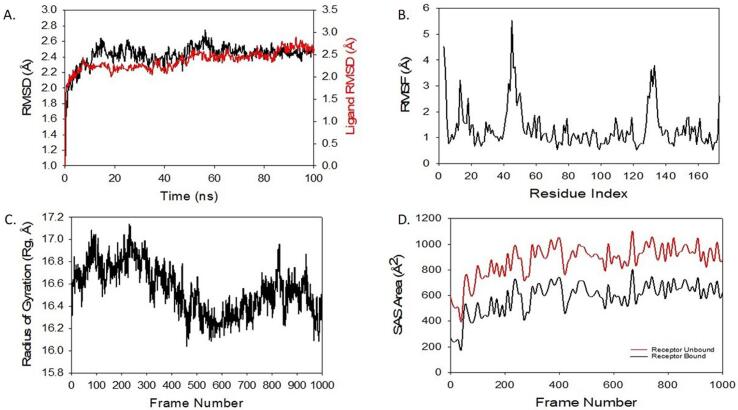
RMSD plots of Bcl2 + paclitaxel, where black plot is of protein and red plot is for ligand. (B) RMSF of Cα backbone of Bcl2 bound to Paclitaxel-ligand, (C) Radius of gyration (Rg) of Cα backbone of Bcl2 bound to Paclitaxel-ligand (D) Solvent accessible surface area of Bcl2 bound to Paclitaxel-ligand. (For interpretation of the references to colour in this figure legend, the reader is referred to the web version of this article.)

Molecular dynamics (MD) and simulation studies were carried out to determine the stability and convergence of Bcl2  + Paclitaxel complex. Simulation of 100 ns displayed stable conformation while comparing the root mean square deviation (RMSD) values. The RMSD of Cα-backbone of Bcl2 bound to Paclitaxel-ligand exhibited a deviation of 2.4 Å (Fig. 1A). While, the Paclitaxel-ligand showed RMSD 2.7 Å **(**Fig. 10 **A).** A stable RMSD plot during simulation signify a good convergence and stable conformations. Therefore, it can be suggested that Bcl2 + Paclitaxel is quite stable in complex due to higher affinity of the ligand. The plot for root mean square fluctuations (RMSF) indicates the residual fluctuations due to conformational variations into different secondary structures. Here RMSF plot displayed fluctuating residues while high fluctuations observed among 17, 50 and 130 residual positions **(**Fig. 10 **B).** The highest fluctuating peaks might be due to higher ordered flexibility conforming into loops **(**Fig. 10 **B).** While no significant fluctuating peaks are observed in case of   Bcl2 + paclitaxel except at 17–20 residual positions**.** This signifies that the Paclitaxel bound Cα-atoms of Bcl2  has significant rigidity. Radius of gyration (Rg) is the measure of compactness of the protein. Here in this study, Bcl2 Cα-backbone bound to Paclitaxel ligand displayed lowering of radius of gyration (Rg) from 16.6 to 16.3 Å **(**Fig. 10 **C).** Significantly lowering gyration (Rg) indicates highly compact orientation of the protein in ligand bound state. Interestingly, we haven’t noticed any interaction of hydrogen bonds between the ligand and binding site residues of protein. Solvent accessible surface area (SASA) in both ligand bound and unbound state was explored. It is clearly visible from the Fig. 10D, that in the unbound state of Paclitaxel to receptor the protein Bcl2 displayed high surface area accessible to solvent in all the cases **(**Fig. 10 **D).** The SASA value lowered as compared to unbound state while bound with ligand **(**Fig. 10 **D).**

### 3.10 Molecular mechanics generalized born surface area (MM-GBSA) calculation

Utilizing the MD simulation trajectory, the binding free energy along with other contributing energy in form of MM-GBSA is determined for Bcl2 bound to Paclitaxel-ligand complex. The results **(**Table 1**)** suggested that the maximum contribution to ΔGbind in the stability of the simulated complexes were due to ΔGbindCoulomb, ΔGbindvdW, ΔGbindHbond and ΔGbindLipo, while, ΔGbindCovalent and ΔGbindSolvGB contributed to the instability of the corresponding complex. Bcl2 + Paclitaxel complex has significantly higher binding free energies dGbind = -74.85 ± 6.79 **(**Table 1**)**.

**Table 1:** Binding free energy components for the BCL2 + Paclitaxel calculated by MM-GBSA.

**Energies (kcal/mol)**	BCL2 + Paclitaxel
ΔG_bind_	−74.85 ± 6.79
ΔG_bind_Lipo	−26.18 ± 1.04
ΔG_bind_vdW	−56.19 ± 2.18
ΔG_bind_Coulomb	−12.27 ± 6.20
ΔG_bind_H_bond_	−1.93 ± 0.34
ΔG_bind_SolvGB	12.34 ± 3.34
ΔG_bind_Covalent	5.83 ± 4.51

### 3.11 Paclitaxel possesses potent anticancer activity

**Fig. 11:**
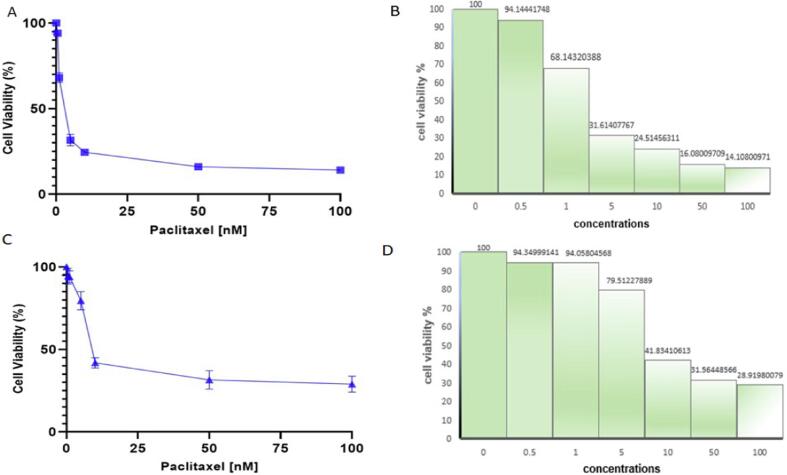
Anti-proliferative effect of paclitaxel on BC cell lines; A) MDA-MB 231 BC cells, C) 4 T1 Cells. The cells were exposed with increased concentrations of PTX. After 72 h, the cell viability was determined by MTT test.

To evaluate the antitumor activity and validate the docking results, a cell viability experiment on MDA-MB-231 and 4 T1 BC cells was done. A 96-well plate with BC cell lines (MDA-MB-231 and 4 T1 cells) was seeded, and paclitaxel was administered in an increased concentration manner. In both the cell lines, the MTT assay demonstrated significant anti-tumor activity of paclitaxel with an IC50 Value of 2.9 nM and 16.4 nM respectively. The low IC50 value reveals the significant effectiveness of paclitaxel in preventing the growth of malignant cells (Fig. 11**).** Additionally, MDA-MB-231 cells demonstrated a significant sensitivity to paclitaxel.

### 3.12 Paclitaxel reduces colony formation in dose dependent manner

**Fig. 12:**
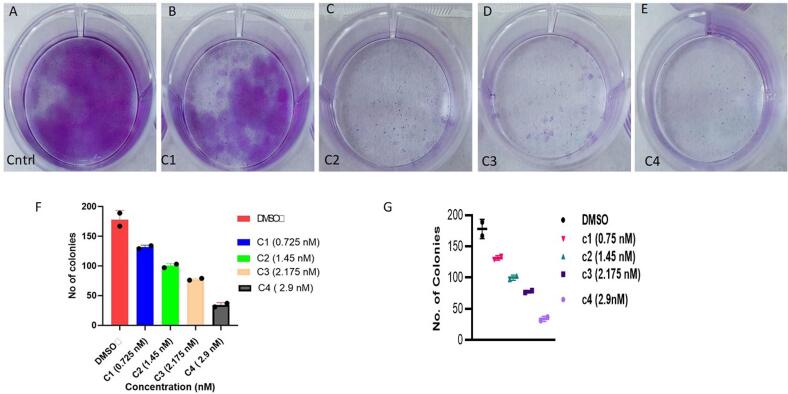
In MDA-MB 231 cells, paclitaxel reduces the colony formation in dose dependent manner. There is a decrease in colony number with increase in the concentration of paclitaxel. Well A treated with no drug shows highest number of colonies, while as well E receiving highest concentration of paclitaxel (c4 = 2.9 nM) shows lowest number of colonies in comparision to other wells.

BC cells (MDA-MB-231) were seeded in 6-well plates at a density of 1,000–1,500 cells per well. The media was changed with a new medium after 48 h, supplemented with increasing concentrations of paclitaxel from 0.725 nM to 2.9 nM **(**Table 2**).** The results of colony formation assay revealed that with increase in concentration of paclitaxel the tendency of colony formation of breast cancer cells decreases and the number was lowest in wells having 2.9 nM paclitaxel **(**Fig. 12**).**

**Table 2:** Various concentrations taken for colony formation assay based on Ic50 value of PTX.

**Concentration**	**No of colonies**
Control (DMSO)	167
C1 = Ic50*0.25 = 0.75 nM	134
C2 = Ic50*0.50 = 1.45Nm	103
C3 = Ic50*0.75 = 2.175 nM	79
C4 = Ic50*1 = 2.9 nM	31

### 3.13 Paclitaxel reduces ROS production, triggering apoptosis of TNBC cells

**Fig. 13:**
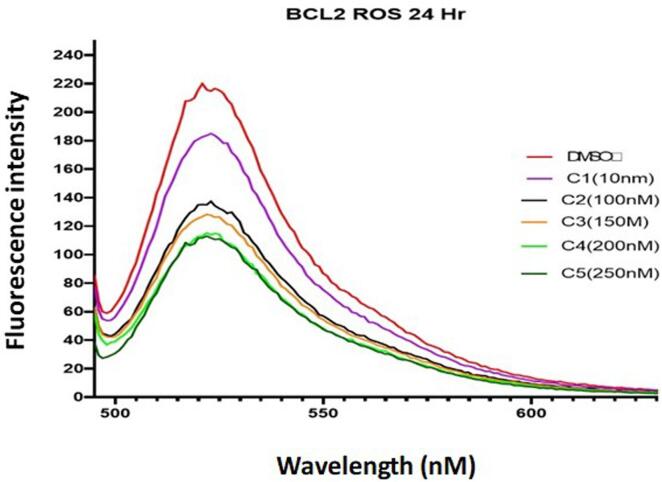
Florescence intensity of ROS levels in MDA-MB 231 BCE cells. The Ros levels are highest in control well and lowest in the highly concentrated paclitaxel well. Therefore, suggesting that Paclitaxel decreases the ROS concentration in MDA-MB 231 BC cells.

The next step was to look at the mechanisms underpinning Bcl2′s anticancer effect. Therefore, following paclitaxel treatment, we evaluated the intracellular ROS levels. The findings showed that paclitaxel dramatically lowers ROS levels in BC cells over the course of 24 h at increasing concentrations. The concentrations were taken in the increasing order from 10 nM to 250 nM. ROS production was seen highest in control well and lowest in the well containing 250 nM treatment **(**Fig. 13**).**

## 4 Discussion

One of the most widespread diseases in women and the 2nd foremost cause of cancer-related fatality globally is BC (Mehraj et al.,). Although improvements in early identification and treatments have increased the OS and RFS of BC individuals, there is an urgent need for attention on the emergence of metastatic and resistant cancers. Exploring novel treatment targets and medication repurposing for existing breast cancer targets is therefore urgently needed (Mir and Mehraj, 2019, Mehraj et al., n.d.). The hallmarks of cancer including angiogenesis, uncontrolled growth and apoptosis circumvention are existing in all tumor cells irrespective of the cause or type. One of the main aim of apoptosis is the inhibition of cancer growth (Tompkins and Thorburn, 2019). The process of “programmed cell death,” sometimes referred to as “apoptosis,” which is a response to environmental stimuli, is useful for treatment and prevention of cancer. Accumulating evidence has revealed that natural products become increasingly important in breast cancer treatment by suppressing cell apoptosis. In light of this, one of the important regulators of apoptosis is Bcl2 family, the inhibition of which can be a significant target for therapy due to the important role it plays in tumorigenesis and treatment resistance. Numerous anticancer medications target the Bcl-2 protein family because it is crucial to cell survival or death. Recent developments in medicine have made it possible to specifically target Bcl-2 family protein–protein interactions (Um, 2016). In mitochondria-mediated apoptosis, members of the Bcl-2 family show both pro- and anti-apoptotic roles. The first proto-oncogene to have a role in suppressing apoptosis and programmed cell death has been identified as Bcl-2. Multiple cell-generated signals related to survival and death are generated by Bcl-2 (Czabotar and Garcia-Saez, 2023). In this study we have used several Insilco methods to analyse the expression pattern of Bcl2 in BC. The work was further extended to target this gene of interest with one of its known inhibitor that is paclitaxel.

The results of TIMER 2.0 analysis illustrate that Bcl2 is overexpressed in several cancers, including HNSC, BC, LUSC, KIRC, LGG, THCA, SARC, LUAD, and UCEC **(**Fig. 1**).** Also, the bc-GenEXMiner was assessed to analyse the link between Bcl2 and clinicopathological traits in BC individuals. The results depicted that the high expression of Bcl2 is associated with HER2^-^, ER^+^, PR^+^, Nodal negative, SBR1, NPI1, wild type P53, and non TNBC status **(**Fig. 2**).** The gene-gene interaction of Bcl2 showed that Bcl2 is highly correlated with Bcl2L11, FKBP8, BBC3 and other genes **(**Fig. 3**).** Using the UALCAN database, expression analysis of Bcl2 in relation to sample type, age group, BC subtypes, and ethnicity was further investigated. The analysis revealed that Bcl2 is overexpressed in primary tumor as compared to the normal patients. The females under the age group of 61–80 show augmented expression of Bcl2. In addition, luminal patients displayed increased Bcl2 expression in comparison to HER2 or TNBC subtypes **(**Fig. 4**).** Furthermore, we analyzed that BC patients with overexpressed Bcl2 show variations in mTOR signaling, WNT signaling, and the p53-Rb pathway **(**Fig. 5**).** Additionally, using TISCH database, the study depicted that Bcl2 is highly upregulated in primary tumors as compared to metastatic tumors. Bcl2 expression depicted heterogeneity in expression profile across the primary cancer cell population **(**Fig. 6**).** The PPI of Bcl2 revealed several genes interacting with Bcl2 and the top 10 hub genes include Bcl2, Bcl2L11, TP53, PMAIP1, BCL2L1, Bcl2L2, Bcl2A1, BIK, BAD, and BAK1 **(**Fig. 7**).**

Bcl2 was further analyzed for gene ontology and KEGG analysis. We analyzed that Bcl2 is highly enriched in intracellular pH elevation, regulation of mitochondrial depolarization, negative regulation of enoikis etc. **(**Fig. 8**A).** Among the BP, Bcl2 was found associated with BH domain binding, protease binding, channel activity, protein heterodimerization activity, etc**. (**Fig. 8**B).** Among the cellular compartment Bcl2 was highly enriched in mitochondrial outer membrane, and nuclear membrane **(**Fig. 8**C).** The KEGG pathway analysis deciphered that Bcl2 is linked with Hedgehog signaling pathway, small cell lung cancer, p53 signalling pathway, prostate cancer, age, race, signalling pathway in diabetic complications, NF-κ β signalling pathway **(**Fig. 8**D).**

The next step was to conduct molecular docking studies to understand how Bcl2 binds to paclitaxel. Paclitaxel strongly bound to protein Bcl2 according to molecular docking studies, with the binding energy being −7.2 kcal/mol **(**Fig. 9**).** Stable RMSD plot suggested that Bcl2 + paclitaxel is quite stable in complex due to higher affinity of the ligand. The RMSF plot displayed fluctuating residues while high fluctuations observed among 17, 50 and 130 residual positions. BCL2 Cα-backbone bound to Paclitaxel ligand displayed lowering of radius of gyration (Rg) from 16.6 to 16.3 Å, which indicates highly compact orientation of the protein in ligand bound state**.** Interestingly, the SASA value lowered as compared to unbound state while bound with ligand **(**Fig. 10**).**

Furthermore, we validated our results of Insilco study using invitro assays. The results of a cell viability MTT test on MDA-MB-231 and 4 T1 cells revealed that paclitaxel possesses significant anti-tumor activity having an IC50 of 2.9 nM and 16.4 nM in MDA-MB-231 and 4 T1 cells, respectively. The low IC50 concentration depicts the high efficiency of paclitaxel in obstructing the tumor cell division **(**Fig. 11**).** The results of colony formation assay revealed that with increase in concentration of paclitaxel the tendency of colony formation of breast cancer cells decreases and the number was lowest in wells having 2.9 nM paclitaxel **(**Fig. 12**).** Also, paclitaxel reduces reactive oxygen species production with increase in its concentration triggering apoptosis of TNBC cells **(**Fig. 13**).** When combined, the study demonstrates that paclitaxel acts as a potent anticancer drug against Bcl2 in BC individuals.

## 5 Conclusion

Our study demonstrated that Bcl2 is overexpressed in BC and possesses oncogenic properties, and targeting Bcl2 with paclitaxel is potent therapeutic strategy to treat breast cancer patients.

## Declaration of competing interest

The authors declare that they have no known competing financial interests or personal relationships that could have appeared to influence the work reported in this paper.
